# Circulating Ketone Bodies, Pyruvate, and Citrate and Risk of Cognitive Decline, Structural Brain Abnormalities, and Dementia

**DOI:** 10.14336/AD.2024.0754

**Published:** 2025-10-13

**Authors:** Laia Gutierrez-Tordera, Kristine F. Moseholm, Marta Trius-Soler, Mònica Bulló, Annette Fitzpatrick, Margery A. Connelly, Oscar L. Lopez, Majken K. Jensen, Marta Guasch-Ferré, Kenneth J. Mukamal

**Affiliations:** ^1^Nutrition and Metabolic Health Research Group, Department of Biochemistry and Biotechnology, Rovira iVirgili University (URV), 43201 Reus, Spain.; ^2^Institute of Health Pere Virgili (IISPV), 43204 Reus, Spain.; ^3^Center of Environmental, Food and Toxicological Technology - TecnATox, Rovira i Virgili University, 43201 Reus, Spain.; ^4^Section for Epidemiology, Department of Public Health, University of Copenhagen, Denmark.; ^5^Novo Nordisk Center for Basic Metabolic Research, University of Copenhagen, Denmark.; ^6^CIBER Physiology of Obesity and Nutrition (CIBEROBN), Carlos III Health Institute, 28029 Madrid, Spain.; ^7^Department of Epidemiology, University of Washington, Seattle, WA, USA.; ^8^Labcorp, Morrisville NC, USA.; ^9^Department of Neurology, University of Pittsburgh, Pittsburgh, PA, USA.; ^10^Department of Nutrition, Harvard T.H. Chan School of Public Health, Boston, MA, USA.; ^11^Department of Medicine, Beth Israel Deaconess Medical Center, Boston, MA, USA.

**Keywords:** ketone body, acetone, pyruvate, citrate, dementia, cognition

## Abstract

The relationship between key energy metabolites and brain health is not well understood. We investigated the association between circulating ketone bodies, pyruvate, and citrate with cognitive decline, structural brain characteristics, and risk of dementia. We measured ketone bodies (acetoacetate, β-hydroxybutyrate, and acetone), pyruvate, and citrate species using NMR in plasma samples from 1,850 older adults in the Cardiovascular Health Study collected in 1989-90 or 1992-93. Cognitive decline was assessed using the modified Mini-Mental State Examination and the Digit Symbol Substitution Test. Dementia was adjudicated by a committee of experts through comprehensive evaluations including cognitive tests, medical records, and interviews with the next of kin. Dementia-related mortality was confirmed by a committee using death certificates and other clinical data from hospitalization. Multivariable linear mixed models were used to assess 9-year cognitive decline, while multivariable Cox regression models evaluated 6-year dementia incidence and 22-year dementia-related mortality. White matter lesions and ventricular size were measured using MRI in 1992-94 and were analyzed using multivariable linear regression models. Higher plasma levels of ketones, particularly β-hydroxybutyrate, were associated with faster cognitive decline (β, -0.10; 95% CI, -0.15 to -0.05; P_adj_<.001) and dementia-related mortality (HR per SD, 1.29; 95% CI, 1.07 to 1.56; P_adj_=0.023). Higher pyruvate concentrations were associated with slower cognitive decline, smaller ventricular size, lower dementia risk (HR per SD, 0.87; 95% CI, 0.77 to 0.97; P=0.013; P_adj_=0.073), and lower dementia mortality. Higher citrate levels were associated with less cognitive decline and lower dementia risk. In adults aged 65 years and older, circulating ketone bodies are associated with faster cognitive decline and higher dementia mortality, while pyruvate and citrate are associated with lower dementia risk.

## INTRODUCTION

Dementia, characterized by a gradual decline in cognitive abilities that interferes with daily life, continues to grow dramatically, with an expected 139 million people affected in 2050 [1]. The increasing global prevalence of dementia highlights the urgency of identifying peripheral biomarkers for high-risk patients to drive effective preventive and therapeutic strategies to delay its progression.

Cognitive decline and dementia are marked by dysregulation in cerebral energy metabolism [2]. The metabolic rate of glucose consumption is reduced in the brain of dementia patients, a pattern seen years before the onset of cognitive impairment [3]. This reduction leads to disruptions in both glycolysis and the tricarboxylic acid (TCA) cycle, subsequently lowering the levels of key metabolites such as pyruvate and citrate [4]. In response to glucose hypometabolism, the brain shifts its main energy substrate utilization towards ketone bodies (acetone, acetoacetate [AcAc], and β-hydroxybutyrate [BHB]) serving as an alternative fuel source for the TCA cycle [5]. This metabolic adaptation is essential for partially preserving brain function during metabolic challenges.

While extensive research has explored brain glucose consumption [6], the impact of ketones, pyruvate, and citrate on cognitive outcomes has primarily involved cellular and animal models [7,8] or ketogenic diets [9]. In observational studies, higher pyruvate levels were found in the cerebrospinal fluid of patients with Alzheimer’s disease (AD) or vascular dementia (VaD) [10], whereas low levels of citrate in serum were associated with lower cognitive decline over five years [11]. A previous study of the UK Biobank showed a positive association of total and individual circulating plasma levels of ketone bodies and citrate with dementia [12]. However, other analyses of the same cohort have not found associations of circulating ketones and citrate with early pathological signs or the risk of dementia incidence [13]. Of note, the latter had a longer follow-up period than the first (14 versus 12 years), a larger number of participants (274,160 versus 110,655), and a higher incidence of dementia (1.9% versus 1.3%). In the Cardiovascular Health Study (CHS), higher concentrations of ketones were associated with the risk of incident heart failure and all-cause mortality [14], but the potential relation with cognitive outcomes and brain structure has not yet been investigated. To our knowledge, only an interventional study using oral intake of medium-chain triglycerides has investigated the effect of ketone bodies on brain structural measures [15].

Therefore, we examined the role of ketone bodies, pyruvate, and citrate as potential risk factors for cognitive decline, incident dementia, and dementia-specific mortality in the CHS, a population-based ongoing longitudinal study of older adults. Given that neurodegeneration progresses with structural brain alterations, we further investigated their associations with white matter lesions and ventricular atrophy.

## MATERIALS AND METHODS

### Study Population

The CHS is a prospective observational multicenter cohort study of cardiovascular disease and risk factors among adults aged 65 years and older from four US countries. The study design and data collection methodology have been previously described elsewhere [16]. Briefly, participants were sampled randomly from Medicare eligibility lists. Eligibility criteria included being aged ≥65 years, planning to remain in the area for the next three years, and not being institutionalized, wheelchair-bound in the home, or undergoing cancer treatment. All participants completed standardized questionnaires assessing medical history at enrolment and underwent a clinic and laboratory examination. There were 5,201 eligible participants recruited in 1989-1990, and an additional 687 predominantly African-American participants in 1992-1993. Annual follow-up clinic visits with interim telephone calls took place from 1989 to 1999, and with bi-annual telephone calls until the present.

**Figure 1.**
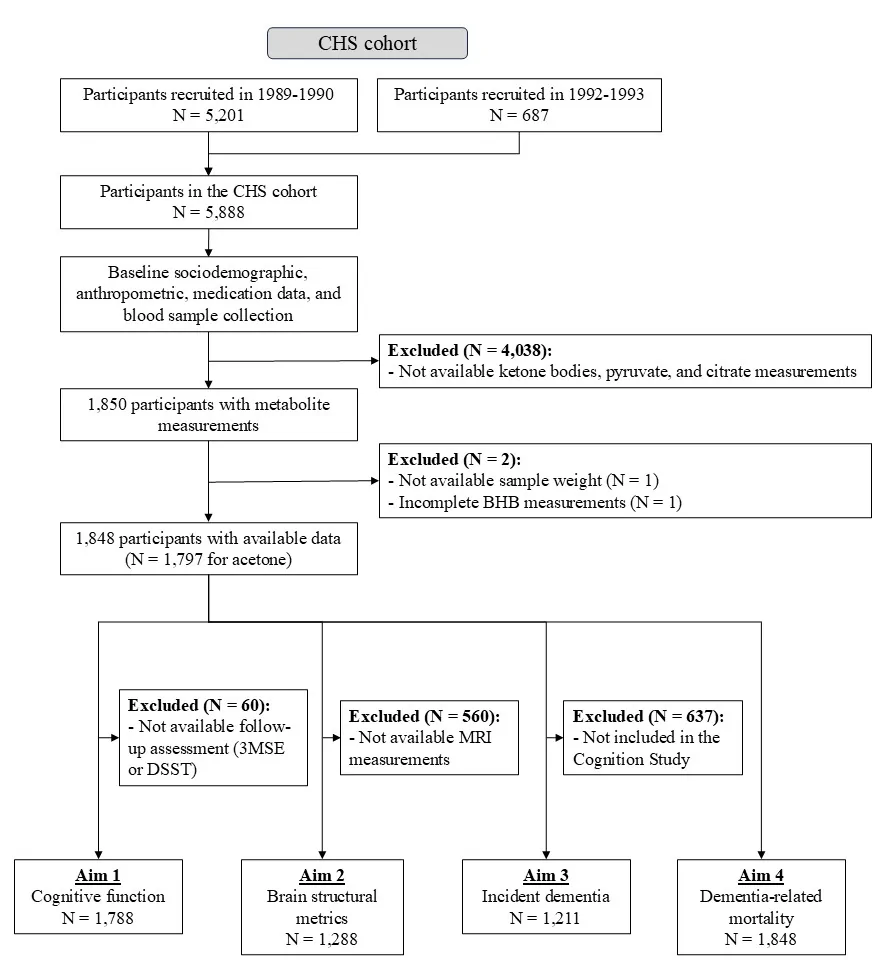
**Flowchart of the study participants**. Abbreviations. CHS, cardiovascular health study; BHB, β-hydroxybutyrate; 3MSE, modified mini-mental state examination; DSST, digit symbol substitution test; MRI, magnetic resonance imaging.

Baseline fasting EDTA-plasma samples from a total of 1,850 participants were sent for nuclear magnetic resonance (NMR) spectroscopy, as previously described [17]. To account for the stratified sampling used in the NMR subset, a sample weight was calculated for each participant. From the total, we evaluated participants for four dementia-related outcomes (Fig. 1). For the primary aim of assessing the association between energy metabolites and cognitive decline, we studied all participants from these 1,850 with at least one follow-up cognitive assessment using the Modified Mini-Mental State Examination (3MSE) or the Digit Symbol Substitution Test (DSST). Second, to investigate the association between the energy metabolites and structural brain abnormalities, participants with available brain magnetic resonance imaging (MRI) were included. Third, to examine the association with incident dementia, we evaluated participants who were followed as part of the CHS Cognition Study, which included all participants who were free of dementia in 1992-1993, underwent MRI, had a cognitive test measured in the same year, and were subsequently adjudicated with dementia, mild cognitive impairment (MCI), or remained cognitively healthy through 1998-1999 [18]. Lastly, we evaluated dementia-related mortality among all participants, using centrally adjudicated causes of death [19]. The analytical dataset was downloaded on January 22, 2024.

### Measurement of Plasma Metabolites

Fasting EDTA-plasma samples were collected in 1989-1990 for the original cohort and 1992-1993 for the new cohort and stored at -70ºC at the CHS Central Laboratory at the University of Vermont [20]. They were shipped to LipoScience (now Labcorp, Morrisville, NC) in 2000 for NMR spectroscopy analysis. Later, employing newly validated algorithms to interrogate the digitized and stored NMR spectra from 2000, the concentrations of ketone bodies, pyruvate, and citrate were calculated in mmol/L [21,22]. Metabolite concentrations are shown in Table 1.

**Table 1:** Baseline characteristics of all study participants according to outcomes (cognitive decline, brain structure, incident dementia, and dementia-related outcomes).

	Cognitive function	Brain structural metrics	Incident dementia	Dementia-related mortality
**N**	1788	1288	1211	1848
**Age (years)**	71 (68, 76)	71 (68, 75)	71 (68, 74)	71 (68, 76)
**Women [N (%)]**	1064 (59.5)	768 (59.6)	729 (60.2)	1096 (59.3)
**Race [N (%)]**				
**White**	1494 (83.6)	1083 (84.1)	1025 (84.6)	1536 (83.1)
**Other**	294 (16.4)	205 (15.9)	186 (15.4)	312 (16.9)
**Education (years)**	12 (11, 18)	12 (11, 19)	12 (12, 19)	12 (11, 18)
**Combined family income (dollars per year)**				
**Low (0 to 11,999)**	412 (23.0)	266 (20.7)	237 (19.6)	432 (23.4)
**Medium (12,000 to 34,999)**	871 (48.7)	646 (50.2)	619 (51.1)	896 (48.5)
**High (>35,000)**	395 (22.1)	300 (23.3)	288 (23.8)	401 (21.7)
**NA**	110 (6.2)	76 (5.9)	67 (5.5)	119 (6.4)
**BMI (kg/m^2^)**	26.1 (23.6, 29.1)	25.9 (23.4, 28.5)	25.9 (23.5, 28.6)	26.1 (23.6, 29.1)
**Weight (kg)**	71.4 (61.9, 82.1)	70.5 (61.2, 80.7)	70.8 (61.9, 80.8)	71.6 (61.9, 82.1)
**APOE ε4 [N (%)]**				
**Carrier**	375 (21.0)	270 (21.0)	249 (20.6)	389 (21.0)
**Non-carrier**	1222 (68.3)	894 (69.4)	853 (70.4)	1261 (68.2)
**NA**	191 (10.7)	116 (9.0)	107 (8.8)	198 (10.7)
**Alcohol (beverages/week)**	0.02 (0, 1.3)	0.02 (0, 1.4)	0.02 (0, 1.5)	0.02 (0, 1.25)
**Smoking [N (%)]**				
**Never**	863 (48.3)	631 (49.0)	587 (48.5)	894 (48.4)
**Former**	719 (40.2)	517 (40.1)	494 (40.8)	740 (40.0)
**Current**	206 (11.5)	140 (10.9)	130 (10.7)	214 (11.6)
**Physical activity (kcal/wk)**	1080 (405, 2385)	1133 (450, 2483)	1142 (480, 2492)	1080 (390, 2340)
**CRP (mg/L)**	2.4 (1.2, 4.3)	2.2 (1.1, 3.9)	2.2 (1.1, 3.8)	2.4 (1.2, 4.3)
**Albumin (g/dL)**	4.0 (3.8, 4.2)	4 (3.8, 4.2)	4.0 (3.8, 4.2)	4.0 (3.8, 4.2)
**Fasting time [N (%)]**				
<**8 hours**	21 (1.2)	14 (1.1)	12 (1.0)	22 (1.2)
> **8 hours**	1697 (95.0)	1218 (94.6)	1145 (94.5)	1753 (94.9)
**NA**	70 (3.9)	56 (4.3)	54 (4.5)	73 (4.0)
**Hypertension**				
**Normotensive**	768 (43.0)	604 (46.9)	579 (47.8)	787 (42.6)
**Borderline**	283 (15.8)	204 (15.8)	188 (15.5)	293 (15.9)
**Hypertensive**	736 (41.2)	480 (37.3)	444 (36.7)	767 (41.5)
**Any diabetes**	264 (14.8)	175 (13.6)	159 (13.1)	281 (15.2)
**Acetone (mmol/L)**	0.013 (0.008, 0.027)	0.013 (0.008, 0.020)	0.013 (0.007, 0.020)	0.013 (0.008, 0.021)
**Acetoacetate (mmol/L)**	0.036 (0.021, 0.062)	0.035 (0.021, 0.061)	0.035 (0.021, 0.060)	0.036 (0.021, 0.063)
**B-hydroxybutyrate (mmol/L)**	0.101 (0.069, 0.161)	0.097 (0.067, 0.153)	0.095 (0.067, 0.152)	0.101 (0.069, 0.161)
**Pyruvate (mmol/L)**	0.050 (0.031, 0.071)	0.050 (0.032, 0.071)	0.050 (0.032, 0.070)	0.050 (0.032, 0.071)
**Citrate (mmol/L)**	0.147 (0.129, 0.169)	0.146 (0.128, 0.167)	0.145 (0.128, 0.167)	0.147 (0.130, 0.169)

NOTE. Continuous data are presented as median (Q1-Q3), and categorical variables are presented as number (%). Data was calculated for participants with complete measurements for acetoacetate, β-hydroxybutyrate, pyruvate, and citrate. ABBREVIATIONS. BMI, body mass index; CRP, C-reactive protein.

### Cognitive Decline, Structural Metrics, and Clinical Outcomes

Participants were assessed annually for cognitive decline from 1989-1990 to 1998-1999 using the 3MSE and the DSST. Initially, the MMSE was administered in 1989-1990, but the 3MSE was used in subsequent years. Consequently, we considered the first 3MSE administered in 1990-1991 as the baseline. Participants recruited in 1992-1993 had scores starting that year. Because cognitive decline may affect clinic visit attendance, the Telephone Interview for Cognitive Status (TICS) was used for those participants who declined in-person visits. The 3MSE was then estimated from the TICS [23].

Structural brain metrics included white matter grade (WMG) and ventricular size measured by MRI in 1992-1994. Briefly, MRI was performed on General Electric or Picker 1.5 Tesla scanners at three of the four field centers and on a 0.35 Tesla Toshiba instrument at the fourth center. The scanning protocol included sagittal T1-weighted localized images and axial spin-density and T2-weighted images, all with 5-mm thickness and no interslice gaps. Imaging data were interpreted by trained neuroradiologists directly from a PDS-4 digital workstation (Vortech, Texas) consisting of four monitors capable of displaying all images simultaneously. Interpretations of the images included parameters for lesion number, size, signal intensity, and anatomic location. Ventricular size refers to the size of the lateral ventricles estimated from the T1-weighted axial images on a scale from 0 to 9 based on a reference standard. A grade of 0 was considered to be slit-like ventricles, while a grade of 9 was very enlarged ventricles. WMG refers to the reading of white matter hyperintensities, with grade 0 being no white matter hyperintensities and 9 almost all white matter involved. Further details of the MRI techniques have been previously described [24-26].

Participants were evaluated for dementia as part of the CHS Cognition Study. Dementia diagnosis and year of onset was adjudicated by a committee of neurologists and psychiatrists using all available information for each participant, including CHS cognitive test scores, primarily data from the 3MSE, DSST, Benton Visual Retention Test, and the modified version of the Center for Epidemiology Studies Depression Scale scores. The diagnostic procedure also included relevant information such as findings in MRI scans, medications to treat dementia, annual neurologic examinations, and hospital records. Participants diagnosed with dementia at the study’s onset were assigned as prevalent dementia. Detailed methodology for the evaluation of dementia has been published [18, 27]. Dementia-specific mortality was adjudicated using available data from medical records, diagnostic reports, laboratory results, death certificates, and interviews with the next of kin. Dementia was adjudicated as the cause of death when it directly caused death, contributed to death, or when no other apparent cause besides dementia was identified. Further information has been reported previously [16,19].

### Covariates

Data used for covariate adjustment was collected at the respective baseline visit. Standardized questionnaires were administered to collect information on age, sex, race, education, combined family income per year, alcohol consumption (number of drinks per week), smoking habit, and physical activity (total kilocalories expended daily). Hypertension status was defined by systolic pressure ≥140, diastolic blood pressure ≥90, or use of antihypertensive medication plus physician diagnosis of hypertension. Diabetes was based on blood glucose or the use of diabetes medication, whereas dysglycemia was defined as having a diagnosis of diabetes, taking medication for diabetes, or having fasting glucose levels above 100 mg/dL. BMI was calculated as measured weight in kilograms divided by measured height in meters squared [16]. Blood biomarkers (C-reactive protein [CRP], albumin) [20] were assessed using standardized methods, and APOE ε4 genotyping was done using the method of Hixson and Vernier [28]. We defined suspected MCI at baseline as a score <88 on the 3MSE [29]. Fasting status was categorized using an 8-hour cutoff, including a category for missing values.

### Statistical Analysis

Characteristics of the study population for the analyses on cognitive decline, brain structural metrics, and incident dementia are shown as the median and interquartile range for quantitative variables, and percentages for categorical variables. The distribution of variables was assessed using the Anderson-Darling normality test. To facilitate comparison across different variables, energy metabolites (ketones, pyruvate, and citrate) concentrations were log-transformed and converted to their z-score by dividing them by their respective standard deviations (SD). We assessed both the overall impact of the ketone pathway on the outcome and the individual effect of each of them to identify specific actions. To do this, we computed a score for ketones by summing the concentrations of the individual ketone bodies. Correlations among ketones, pyruvate, and citrate were estimated using Spearman correlation coefficients. Sampling weights were computed for every participant within subcohorts to estimate associations that reflect the original CHS cohort. We evaluated four sets of outcomes: cognitive decline, brain structural abnormalities, incident dementia, and dementia-related mortality. The primary analysis comprised weighted mixed-effects linear regression models to examine the independent associations between one SD increase in log-transformed energy metabolite concentration (mmol/L) and cognitive function over time. This was estimated by the annual rate of change in mean 3MSE and DSST scores during follow-up. All available time-points per participant were used. The primary measure of association was the interaction between the metabolites’ concentration and the time from baseline to test evaluation. Covariates were treated as fixed effects and participants as random effects with a compound symmetry covariance structure to account for non-independence in the data. In Model 1, we adjusted for age, sex, race, site of recruitment, education, combined family income, and APOE ε4 carrier status; in Model 2, we further adjusted for baseline BMI, BMI^2^, alcohol consumption, alcohol consumption^2^, cigarette smoking, physical activity, CRP, albumin, fasting time, and prevalent hypertension and diabetes. When ketones were examined separately, Model 3 was considered to mutually adjust the ketones and examine their independent contributions.

For structural MRI findings, we used weighted linear regression models to explore cross-sectional associations of the energy metabolites with WMG or ventricular size at the MRI conducted in 1992-1994. The same models of adjustments as in the analysis of cognitive function were used.

We evaluated the risk of incident dementia using weighted Cox regression models. Time-to-dementia onset was calculated as the time elapsed between the Cognition Study baseline (the MRI conducted in 1992-1994) and the earliest of: onset of dementia, death, end of follow-up in 1999, or loss of follow-up. Model 1 and Model 2 were adjusted for the same covariates as the previous analyses. To address the possibility of participants having MCI at baseline, Model 3 was further adjusted for baseline MCI (based on 3MSE). In the analysis of individual ketones, Model 4 additionally included mutual adjustment of the ketone body species.

Lastly, we assessed the association between ketones, pyruvate, and citrate and the risk of dementia-related mortality using weighted Cox regression models. Time-to-death was calculated as the time elapsed between baseline and dementia-specific death, other causes of death, end of follow-up in 2015, or loss of follow-up. We used the same models as in the cognitive decline analyses. Since circulating energy metabolite levels may vary over time, relying on single metabolites to predict long-term outcomes, such as dementia-related mortality during the extended follow-up period in the CHS, presents a significant challenge. Thus, we also performed a sensitivity analysis that restricted follow-up to 15 years using a weighted Cox regression model. We adjusted for the same confounders as in the dementia-related mortality analyses. Additionally, given that some participants did not fast for a minimum of 8 hours, we also repeated all the previous analyses but excluded participants with <8 hours of fasting. To better understand the trajectories of the metabolites in relation to cognitive decline over aging, we checked potential interactions between each of the metabolites and age on each of the dementia-related outcomes included in this study. We also investigated potential interactions between metabolites and dysglycemia on all dementia-related outcomes tested. P values were adjusted (P_adj_) for multiple testing using the false discovery rate approach (Benjamini-Hochberg method), and significance was set at Padj<.05. Missing data were excluded from the analyses. All analyses were conducted using R software (v. 4.3.0) (R Foundation for Statistical Computing, Vienna, Austria).

### Standard Protocol Approvals, Registrations, and Patient Consent

All procedures followed the Helsinki Declaration of 1975, each site-specific institutional review board approved the study, and all study participants provided voluntary written informed consent.

### Data Availability

Further anonymized data will be provided under request and with an appropriately signed data distribution agreement at www.chs-nhlbi.org.

## RESULTS

The baseline characteristics across the study populations were similar (Table 1). Of the 1,850 participants, 1,848 had available sample weights and complete metabolite measurements for AcAc, BHB, pyruvate, and citrate, while 1,797 had metabolite data for acetone. From the total sample population, 1,788 had two or more measurements taken in the 3MSE or the DSST, 1,288 had brain structural metrics measured, 1,211 were assessed for dementia in the Cognition Study, and all the 1,848 participants were evaluated for dementia-mortality (Fig. 1). The median age of the participants was 71 years old, 59.3-60.2% were women, 83.1-84.6% were White individuals, all had a median of 12 years of education, and 20.6-21% were APOE ε4 carriers. Sex-specific differences for the cognitive function sample population are detailed in Supplementary Table 1.

The degree of correlation varied widely among energy metabolites, with the highest correlations between the ketone bodies, AcAc and BHB (ρ=0.77), and AcAc and acetone (ρ=0.43), as expected. The lowest correlations occurred between BHB and pyruvate (ρ=-0.26) (P<.001 for all values) (Supplementary Fig. 1). In addition, ketones were correlated with age (ρ=0.15; P<.001).

### Cognitive decline

Estimates representing the annual change in cognitive testing per SD log metabolite are shown in Table 2. Higher baseline ketone levels were associated with a faster decline in cognitive function, while higher pyruvate and citrate levels were associated with a slower decline. In a fully covariate-adjusted model (Model 2), ketone body levels were independently associated with greater cognitive decline on the 3MSE test. This pattern was strongest for BHB (β, -0.10; 95% CI, -0.15 to -0.05; P_adj_<.001) but also observed for AcAc (β, -0.09; 95% CI, -0.15 to -0.02; P_adj_=0.018) (Model 3). In contrast, higher pyruvate and citrate levels were associated with less cognitive decline on the 3MSE, which in the case of pyruvate was also observed for the DSST. Ketones and pyruvate, but not citrate, remained significant after correcting for multiple comparisons.

**Table 2:** Association of ketone bodies, pyruvate, and citrate, and cognitive function score trajectories through year 11

Test	Metabolite	Model 1 β (95% CI)	P value	P_adj_	Model 2 β (95% CI)	P value	P_adj_	Model 3 β (95% CI)	P value	P_adj_
3MSE	Ketone bodies score	-0.081 (-0.134, -0.027)	0.003**	0.011*	-0.079 (-0.132, -0.025)	0.004**	0.012*	NA	NA	NA
	**Acetone**	0.031 (-0.023, 0.084)	0.259	0.299	0.025 (-0.029, 0.078)	0.367	0.371	0.024 (-0.029, 0.078)	0.371	0.371
	**AcAc**	-0.056 (-0.112, -0.001)	0.046*	0.063	-0.052 (-0.107, 0.003)	0.063	0.079	-0.086 (-0.148, -0.023)	0.007**	0.018*
	**BHB**	-0.096 (-0.149, -0.042)	<0.001***	<0.001***	-0.095 (-0.148, -0.042)	<0.001***	<0.001***	-0.099 (-0.153, -0.046)	<0.001***	<0.001***
	**Pyruvate**	0.056 (0.007, 0.105)	0.026*	0.049*	0.062 (0.013, 0.111)	0.013*	0.028*	NA	NA	NA
	**Citrate**	0.038 (0.002, 0.073)	0.039*	0.063	0.036 (0.001, 0.072)	0.045*	0.063	NA	NA	NA
DSST	Ketone bodies score	0.005 (-0.037, 0.047)	0.811	0.885	0.006 (-0.036, 0.048)	0.774	0.885	NA	NA	NA
	**Acetone**	0.016 (-0.025, 0.057)	0.441	0.662	0.018 (-0.023, 0.058)	0.400	0.662	0.017 (-0.024, 0.058)	0.415	0.662
	**AcAc**	0.031 (-0.014, 0.077)	0.174	0.522	0.032 (-0.013, 0.078)	0.158	0.522	0.049 (0, 0.099)	0.052	0.260
	**BHB**	-0.005 (-0.047, 0.037)	0.817	0.885	-0.004 (-0.046, 0.038)	0.844	0.885	-0.003 (-0.045, 0.039)	0.885	0.885
	**Pyruvate**	0.074 (0.033, 0.115)	<0.001***	<0.001***	0.078 (0.037, 0.119)	<0.001***	<0.001***	NA	NA	NA
	**Citrate**	0.018 (-0.013, 0.049)	0.244	0.546	0.018 (-0.013, 0.049)	0.255	0.546	NA	NA	NA

NOTE. β represents the metabolite:time interaction estimate (score change per year per standard deviation log metabolite). Model 1 was adjusted for age, sex, race, recruiting center, educational level, combined family income, and APOE ε4; Model 2 further adjusted for physical activity, BMI, BMI^2^, alcohol intake, alcohol intake^2^, fasting time, C-reactive protein, albumin, hypertension and diabetes diagnosis; Model 3 additionally included mutual ketone body adjustment. FDR was applied to correct for multiple comparisons (P_adj_). *P value <.05, **P value <.01, ***P value <.001. ABBREVIATIONS. 3MSE, modified mini-mental state examination; DSST, digit symbol substitution test; AcAc, acetoacetate, BHB, B-hydroxybutyrate; FDR, false discovery rate; CI, confidence interval; BMI, body mass index.

### Structural brain findings

**Figure 2.**
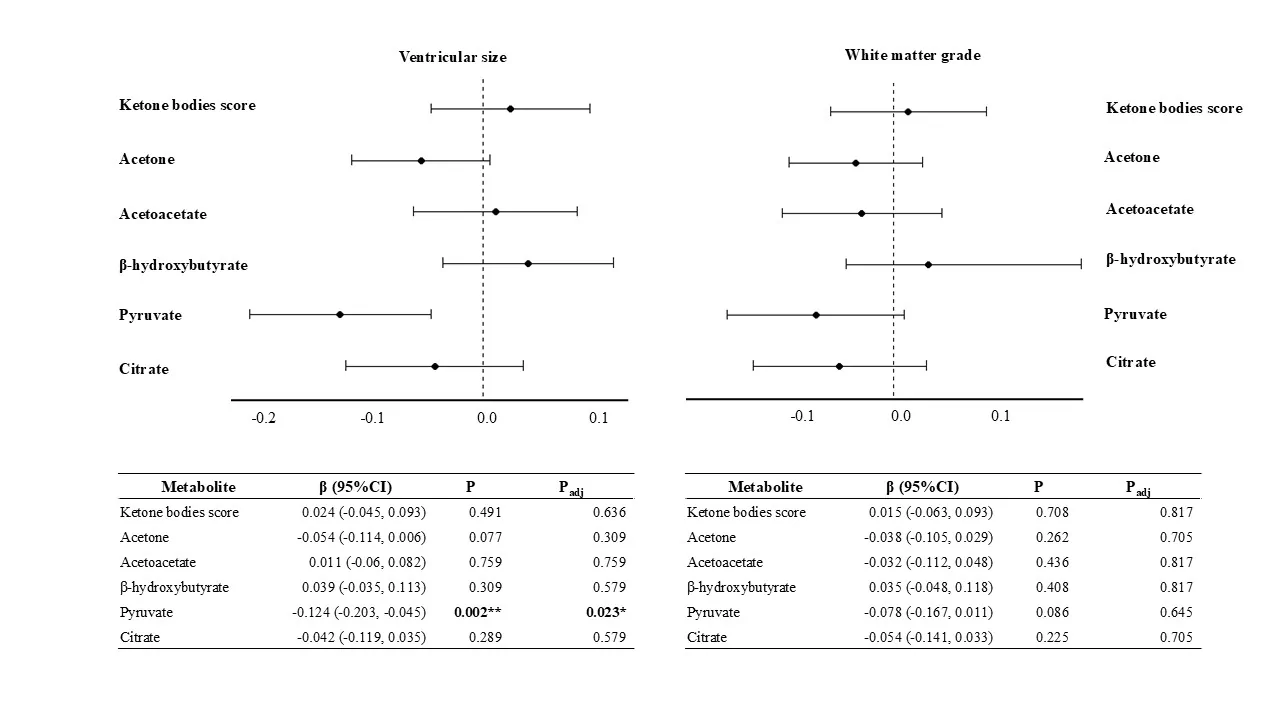
**Association of ketone bodies, pyruvate, and citrate, with white matter grade and ventricular grade**. NOTE. β coefficients for score change in white matter grade or ventricular grade per-standard deviation log metabolite concentrations. Model adjusted for age, sex, race, recruiting center, educational level, combined family income, APOE ε4, physical activity, BMI, BMI^2^, alcohol intake, alcohol intake^2^, fasting time, C-reactive protein, albumin, hypertension and diabetes diagnosis, and mutual adjustment of ketone body species. FDR was applied to correct for multiple comparisons (P_adj_). **P value <.01, *P value <.05. ABBREVIATIONS. CI, confidence interval; BMI, body mass index; FDR, false discovery rate.

In analyses of WMG and ventricular size (where higher scores indicate more structural damage), pyruvate was inversely associated with ventricular size (β, -0.12; 95% CI, -0.20 to -0.05; P_adj_=0.023; Fig. 2). In comparison, no apparent relationship was noted between pyruvate and WMG (Supplementary Table 2). We found no evidence of associations of ketone bodies or citrate with either ventricular size or WMG.

### Incident dementia in the CHS Cognitive Study

**Figure 3.**
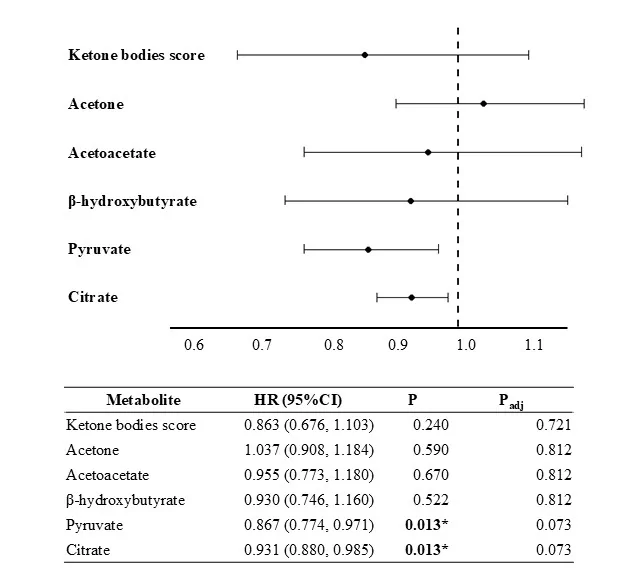
**Association of ketone bodies, pyruvate, and citrate levels with incident dementia risk**. NOTE. HR for incident dementia per standard deviation log metabolite concentrations. Model adjusted for age, sex, race, recruiting center, educational level, combined family income, APOE ε4, physical activity, BMI, BMI^2^, alcohol intake, alcohol intake^2^, fasting time, C-reactive protein, albumin, hypertension and diabetes diagnosis, suggested MCI condition, and mutual adjustment of ketone body species. FDR was applied to correct for multiple comparisons (P_adj_). *P value <.05. ABBREVIATIONS. CI, confidence interval; BMI, body mass index; FDR, false discovery rate.

Among the subset of 1,211 participants in the CHS Cognitive Study who also had metabolites assayed, 184 incident dementia cases occurred during a median of 6 years of follow-up. Pyruvate and citrate were each associated with a lower risk of incident dementia in fully adjusted models (Model 3) (Fig. 3), consistent with the findings observed for cognitive decline in the larger cohort. Pyruvate had the strongest association with dementia incidence, with a 13.3% lower risk (HR per SD, 0.87; 95% CI, 0.78 to 0.97; P=0.013; P_adj_=0.073) compared to a 6.9% for citrate (HR per SD, 0.93; 95% CI, 0.88 to 0.99; P=0.013; P_adj_=0.073; Supplementary Table 3).

### Dementia-specific mortality

The Table 3 shows the associations of ketones, pyruvate, and citrate levels with dementia-related mortality over 22 years of follow-up. Among a total of 1,848 participants, 250 had dementia adjudicated as the cause of death. Ketone bodies were associated with a higher risk of dementia-related mortality, mainly driven by BHB (HR per SD, 1.29; 95% CI, 1.07 to 1.56; P_adj_=0.023). Higher pyruvate levels also tended to be associated with a lower risk of dementia mortality. In sensitivity analysis, censoring the follow-up at 15 years strengthened the observed association of BHB and demonstrated an association of acetone with dementia-related mortality independently of the other ketones (HR per SD, 0.79; 95% CI, 0.66 to 0.94; P_adj_=0.022) (Table 4).

### Sensitivity Analyses

In sensitivity analyses, the associations of ketone bodies, pyruvate, and citrate remained after excluding participants with unknown or less than 8 hours of fasting time. For pyruvate, the association with 3MSE and dementia mortality was attenuated. For citrate, associations were attenuated in the incident dementia analysis (Supplementary Tables 4 to 7). In other sensitivity analyses, we observed the effect of metabolites on cognitive function (3MSE) to be modified by age, but in stratification analyses, the significant associations were no longer nominally significant, although the attenuation appeared attributable to the smaller sample size within each age group (Supplementary Table 8). Instead, dysglycemia did not seem to modify the effect of the metabolites on the dementia-related outcomes evaluated.

## DISCUSSION

In this large prospective study of deep-phenotyped US adults aged 65 and older, baseline plasma ketone levels were associated with greater cognitive decline and risk of dementia-related mortality, whereas pyruvate and citrate levels were associated with less cognitive decline and lower dementia risk. Pyruvate was also inversely related to ventricular size and risk of dementia mortality.

In cognitively impaired individuals, the deficit in brain glucose utilization is on the order of 20-25% compared to age-matched healthy individuals [30]. To preserve energy-requiring activity, the brain can use ketone bodies. In our study, pyruvate levels were inversely correlated with ketone body levels, suggesting a potential shift in mitochondrial fuel metabolism. In addition, given that BHB and acetone are derived from AcAc [4], strong associations between the three species were observed.

### Ketone bodies

In hippocampal neurons exposed to Aβ_1-42_, ketone bodies have been shown to provide an alternative source of mitochondrial acetyl CoA. In observational analyses of 5,506 participants aged ≥ 45 years from the Whitehall II study, BHB was positively associated with cognitive function and a lower risk of AD incidence [31]. Similarly, the administration of exogenous ketone bodies has suggested a beneficial effect on cognitive decline [32]. In contrast, in a prospective study of 110,655 participants from the UK Biobank, ketone body levels were positively associated with dementia incidence [12], whereas in a larger study of the same cohort, they showed no association with the risk of incident dementia [13]. In our study, ketone bodies were associated with faster cognitive decline. We hypothesize these discrepancies might arise due to differences in ketone body concentrations (BHB levels reached 0.32 mmol/L in the Whitehall II study but 0.10 mmol/L in our study, no data is provided for the UK Biobank), age differences (the median age was 55 years in the Whitehall II study, 56 in the UK Biobank, and 71 years in the CHS population) or diagnosis criteria (in the UK Biobank, for example, the diagnosis was based on hospital ICD codes, which entails a high number of missing cases and late diagnosis [33]). We did not observe associations with white matter lesions or ventricular atrophy, although, in a female animal model, a decline in plasma ketone bodies was coincident with white matter degeneration [34].

**Table 3:** Association of ketone bodies, pyruvate, and citrate levels with dementia-related mortality.

Metabolite	Model 1 HR (95% CI)	P value	P_adj_	Model 2 HR (95% CI)	P value	P_adj_	Model 3 HR (95% CI)	P value	P_adj_
**Ketone bodies score**	1.377 (1.173, 1.617)	<0.001***	<0.001***	1.279 (1.075, 1.521)	0.005**	0.020*	NA	NA	NA
**Acetone**	1.035 (0.876, 1.224)	0.683	0.788	1.028 (0.868, 1.218)	0.747	0.800	0.992 (0.846, 1.164)	0.925	0.925
**AcAc**	1.232 (0.904, 1.681)	0.187	0.312	1.153 (0.947, 1.404)	0.156	0.292	1.045 (0.903, 1.209)	0.558	0.697
**BHB**	1.395 (1.181, 1.648)	<0.001***	0.001**	1.296 (1.085, 1.547)	0.004**	0.020*	1.289 (1.069, 1.555)	0.008**	0.023*
**Pyruvate**	0.896 (0.782, 1.026)	0.111	0.238	0.852 (0.749, 0.968)	0.014*	0.035*	NA	NA	NA
**Citrate**	1.216 (0.712, 2.076)	0.473	0.646	1.352 (0.816, 2.239)	0.242	0.363	NA	NA	NA

NOTE. Hazard ratios represent risk per standard deviation log (mmol/L) metabolite. Model 1 was adjusted for age, sex, race, recruiting center, education level, combined family income, and APOE ε4; Model 2 further adjusted for physical activity, BMI, BMI^2^, alcohol intake, alcohol intake^2^, fasting time, C-reactive protein, albumin, physical activity, hypertension and diabetes diagnosis; Model 3 additionally included mutual ketone body adjustment. FDR was applied to correct for multiple comparisons (P_adj_). *P value <.05. ABBREVIATIONS. AcAc, acetoacetate; BHB, B-hydroxybutyrate; HR, hazard ratio; CI, confidence interval; BMI, body mass index; FDR, false discovery rate.

In our study, higher plasma levels of ketone bodies, and particularly BHB, were associated with a higher risk of dementia-related mortality after 22 years of follow-up. An increase in plasma ketone bodies can occur with glucose impairment at the cost of fatty acid oxidation, potentially compromising various organ functions and contributing to dementia-related mortality [14, 35]. Notably, after 15 years of follow-up, acetone levels were associated with dementia mortality, which might reflect compensation for the energy deficit in the brain. Therefore, the fluctuating levels of ketone bodies over the progression of the disease pose a challenge when using single metabolites as biomarkers for long-term outcomes.

**Table 4:** Association of ketone bodies, pyruvate, and citrate levels with dementia-related mortality censoring at 15 years.

Metabolite	Model 1 HR (95% CI)	P value	P_adj_	Model 2 HR (95% CI)	P value	P_adj_	Model 3 HR (95% CI)	P value	P_adj_
**Ketone bodies score**	1.538 (1.271, 1.861)	<0.001***	<0.001***	1.357 (1.089, 1.691)	0.007**	0.020*	NA	NA	NA
**Acetone**	0.849 (0.724, 0.997)	0.046*	0.086	0.829 (0.69, 0.995)	0.045*	0.086	0.791 (0.664, 0.942)	0.009**	0.022*
**AcAc**	1.205 (0.665, 2.184)	0.539	0.573	1.089 (0.809, 1.467)	0.573	0.573	0.912 (0.746, 1.114)	0.368	0.424
**BHB**	1.572 (1.288, 1.919)	<0.001***	<0.001***	1.398 (1.126, 1.737)	0.002**	0.009**	1.527 (1.205, 1.935)	<0.001***	0.002**
**Pyruvate**	0.886 (0.703, 1.115)	0.302	0.377	0.872 (0.758, 1.003)	0.055	0.091	NA	NA	NA
**Citrate**	1.459 (0.764, 2.786)	0.252	0.344	1.587 (0.867, 2.903)	0.134	0.201	NA	NA	NA

NOTE. Hazard ratios represent risk per standard deviation log (mmol/L) metabolite. Model 1 was adjusted for age, sex, race, recruiting center, education level, combined family income, and APOE ε4; Model 2 further adjusted for physical activity, BMI, BMI^2^, alcohol intake, alcohol intake^2^, fasting time, C-reactive protein, albumin, physical activity, hypertension and diabetes diagnosis; Model 3 additionally included mutual ketone body adjustment. FDR was applied to correct for multiple comparisons (P_adj_). *P value <.05. ABBREVIATIONS. AcAc, acetoacetate; BHB, B-hydroxybutyrate; HR, hazard ratio; CI, confidence interval; BMI, body mass index; FDR, false discovery rate.

### Pyruvate

There is remarkably limited research investigating the effects of pyruvate on dementia-related outcomes. While ketone body uptake into the brain occurs through monocarboxylate transporters, which remain functional in dementia(36), glucose uptake and the glycolytic pathway are restricted. As a result, lower levels of pyruvate are associated with worse cognitive performance. This was observed in AD cell models, where mitochondrial pyruvate uptake was defective [37]. Aβ_1-42_ has been reported to stimulate the phosphorylation of pyruvate dehydrogenase and inhibit the conversion of pyruvate to acetyl CoA, which is required to fuel the TCA cycle to provide NADH needed to power electron transport and synthesize ATP. When ATP levels are low, mitochondria become unable to pump out calcium, resulting in its accumulation, triggering mitochondrial dysfunction, and promoting apoptosis, increased ROS production, and more aggregation of Aβ [38]. In a rat model of AD, systemic pyruvate administration ameliorated learning and memory [39]. In our study, pyruvate tended to be associated with a lower risk of incident dementia and dementia mortality. In addition, pyruvate has shown neuroprotective effects by targeting hypometabolism, oxidative stress, and neuroinflammation, three hallmarks of ventricular atrophy [39, 40], which could explain higher pyruvate levels in our study associated with lower ventricular atrophy. However, we did not observe an association between pyruvate and the presence of lesions in the white matter. This might be attributed to minimal lesions in the white matter of the study population in CHS with metabolites measured, with 84% of participants having scores ranging from 0 to 3. Additionally, after excluding participants not fasting for eight hours, the effect sizes did not substantially change.

### Citrate

To our knowledge, few studies have examined the association of circulating citrate with cognitive outcomes. In the aforementioned observational study in the UK Biobank, citrate was associated with increased incident dementia [12]. Instead, our findings show a slower cognitive decline and a lower risk of incident dementia for this metabolite. Discrepancies may arise from variations in the adjudication of dementia. The UK Biobank records consist in part of the National Health Service (NHS) in the UK sending updated records for participants to the UK Biobank administration, which then uploads deidentified data to the database for use in applicable projects. Discrepancies between studies might be because of the massive drop-off in sample size from the baseline assessment to subsequent assessments, due to various factors of interest absent in many participants, the exact code included to represent all-cause dementia, or the UK Biobank only asking participants if they had dementia in demographic assessments [33]. In addition, prior evidence *in vitro* has demonstrated the downregulation of the enzyme citrate synthase in patients with AD, and thus of citrate [7], which aligns with our results. When excluding participants who did not meet fasting time criteria, citrate lost its significant association with incident dementia, but the difference in its effect size was not substantial. We did not observe associations of citrate with white matter lesions, ventricular size, or dementia-related mortality.

Previous studies have shown that chronic insulin resistance, a key trait of type 2 diabetes, has been linked to both impaired brain glucose metabolism and increased dementia risk [41]. However, in our study, the effect of the metabolites on dementia-related outcomes did not appear to vary based on dysglycemia status. We suggest that this might be attributed to an insufficient sample size. Additionally, age-related declines in growth hormones, such as IGF-1, which play a role in brain plasticity and cognitive function [42], could promote the metabolic shifts observed in our study.

### Strengths and limitations

This study has several strengths, including its prospective study design, large and well-characterized cohort of older adults, long follow-up, and comprehensive assessment of dementia-related outcomes. However, there are also limitations. First, our associations do not imply causality. Second, the fasting time before blood collection was not the same for all participants, which might have introduced some random variation as shown in sensitivity analyses. Third, dementia-related mortality adjudication might have been incomplete, as participants were indirectly assessed. Fourth, repeating metabolite measurements over time would have provided valuable information. Lastly, the study participants were primarily white, with a roughly proportionate number of Black individuals to the US population, but findings should be interpreted with caution when generalizing to other populations.

### Conclusion

In this study of adults ≥65 years, circulating ketone bodies were associated with cognitive decline and dementia mortality. Additionally, pyruvate and citrate were associated with less cognitive decline and lower risk of incident dementia. Notably, pyruvate was inversely associated with ventricular enlargement and dementia mortality. Novel approaches for preventing and managing cognitive decline and dementia are warranted, and measurements of ketone bodies, pyruvate, and citrate in the blood hold the potential as markers for guiding preventive measures and the design of dietary and therapeutic interventions while offering minimal invasiveness and ready accessibility compared to the current techniques. Furthermore, these metabolites can provide further understanding of the biological mechanisms of neurodegeneration.

## Supplementary Materials

The Supplementary data can be found online at: www.aginganddisease.org/EN/10.14336/AD.2024.0754.
