# Supplementary material for: Circulating Ketone Bodies, Pyruvate, and Citrate and Risk of Cognitive Decline, Structural Brain Abnormalities, and Dementia
Source: Aging Dis. 2024 Oct 13;16(5):3055–68. doi: 10.14336/AD.2024.0754 (PMC12339114; doi:10.14336/AD.2024.0754)
Supplement: Supplementary file 1 — The Supplementary data can be found online at: www.aginganddisease.org/EN/10.14336/AD.2024.0754. [file AD-16-5-3055-s.pdf]

SUPPLEMENTARY DATA

Circulating Ketone Bodies, Pyruvate, and Citrate and Risk of Cognitive Decline, Structural Brain Abnormalities, and Dementia

Laia Gutierrez-Tordera, Kristine F. Moseholm, Marta Trius-Soler, Mònica Bulló, Annette Fitzpatrick, Margery A. Connelly, Oscar L. Lopez, Majken K. Jensen, Marta Guasch-Ferré, Kenneth J. Mukamal

© 2024. Gutierrez-Tordera L. et al. Published online at http://www.aginganddisease.org/EN/10.14336/AD.2024.0754

Supplementary Table 1. Baseline characteristics of the study participants in the cognitive function analysis according to sex.

	Women	*Men*	*Total*
N (%)	1064	724	1788
***Age** (years)*	71 (68, 76)	72 (69, 76)	71 (68, 76)
***Race** [N (%)]*			
White	874 (82.1)	620 (85.6)	1494 (83.6)
Other	190 (17.9)	104 (14.4)	294 (16.4)
***Education** (years)*	12 (11, 18)	12 (11, 20)	12 (11, 18)
***Combined family income** (dollars per year)*			
Low (0 to 11,999)	299 (28.1)	113 (15.6)	412 (23.0)
Medium (12,000 to 34,999)	490 (46.1)	381 (52.6)	871 (48.7)
High (>35,000)	196 (18.4)	199 (27.5)	395 (22.1)
NA	79 (7.4)	31 (4.3)	110 (6.2)
***BMI** (kg/m^2)*	26.1 (23.1, 29.4)	26.2 (24.3, 28.5)	26.1 (23.6, 29.1)
***Weight** (kg)*	65.3 (58.2, 75.0)	79.4 (72.1, 87.1)	71.4 (61.9, 82.1)
***APOE ε4** [N (%)]*			
Carrier	239 (22.5)	150 (20.7)	375 (21.0)
Non-carrier	742 (69.7)	519 (71.7)	1222 (68.3)
NA	115 (10.8)	83 (11.5)	191 (10.7)
***Alcohol** (beverages/week)*	0 (0, 0.6)	0.3 (0, 3.7)	0.02 (0, 1.3)
***Smoking** [N (%)]*			
Never	630 (59.2)	233 (32.2)	863 (48.3)
Former	312 (29.3)	407 (56.2)	719 (40.2)
Current	122 (11.5)	84 (11.6)	206 (11.5)
***Physical activity** (kcal/wk)*	944 (315, 2141)	1303 (543, 2703)	1080 (405, 2385)
***CRP** (mg/L)*	2.4 (1.2, 4.4)	2.3 (1.2, 4.1)	2.4 (1.2, 4.3)
***Albumin** (g/dL)*	4.0 (3.8, 4.2)	4.0 (3.8, 4.2)	4.0 (3.8, 4.2)
***Fasting time** [N (%)]*			
< 8 hours	14 (1.3)	7 (1.0)	21 (1.2)
> 8 hours	1014 (95.3)	683 (94.3)	1697 (95.0)
NA	36 (3.4)	34 (4.7)	70 (3.9)
Hypertension			
Normotensive	458 (43.0)	310 (42.8)	768 (43.0)
Borderline	154 (14.5)	129 (17.8)	283 (15.8)
Hypertensive	452 (42.5)	284 (39.2)	736 (41.2)
Any diabetes	136 (12.8)	128 (17.7)	264 (14.8)
***Acetone** (mmol/L)*	0.012 (0.007, 0.019)	0.015 (0.009, 0.023)	0.013 (0.008, 0.027)
***Acetoacetate** (mmol/L)*	0.034 (0.021, 0.060)	0.039 (0.023, 0.066)	0.036 (0.021, 0.062)
***B-hydroxybutyrate** (mmol/L)*	0.105 (0.071, 0.160)	0.096 (0.067, 0.161)	0.101 (0.069, 0.161)
***Pyruvate** (mmol/L)*	0.050 (0.033, 0.072)	0.049 (0.029, 0.068)	0.050 (0.031, 0.071)
***Citrate** (mmol/L)*	0.150 (0.133, 0.172)	0.143 (0.123, 0.165)	0.147 (0.129, 0.169)

NOTE. Continuous data are presented as median (Q1-Q3), and categorical variables are presented as number (%). Data was calculated for participants with complete measurements for acetoacetate, β-hydroxybutyrate, pyruvate, and citrate. ABBREVIATIONS. BMI, body mass index; CRP, C-reactive protein.

© 2024. Gutierrez-Tordera L. et al. Published online at http://www.aginganddisease.org/EN/10.14336/AD.2024.0754

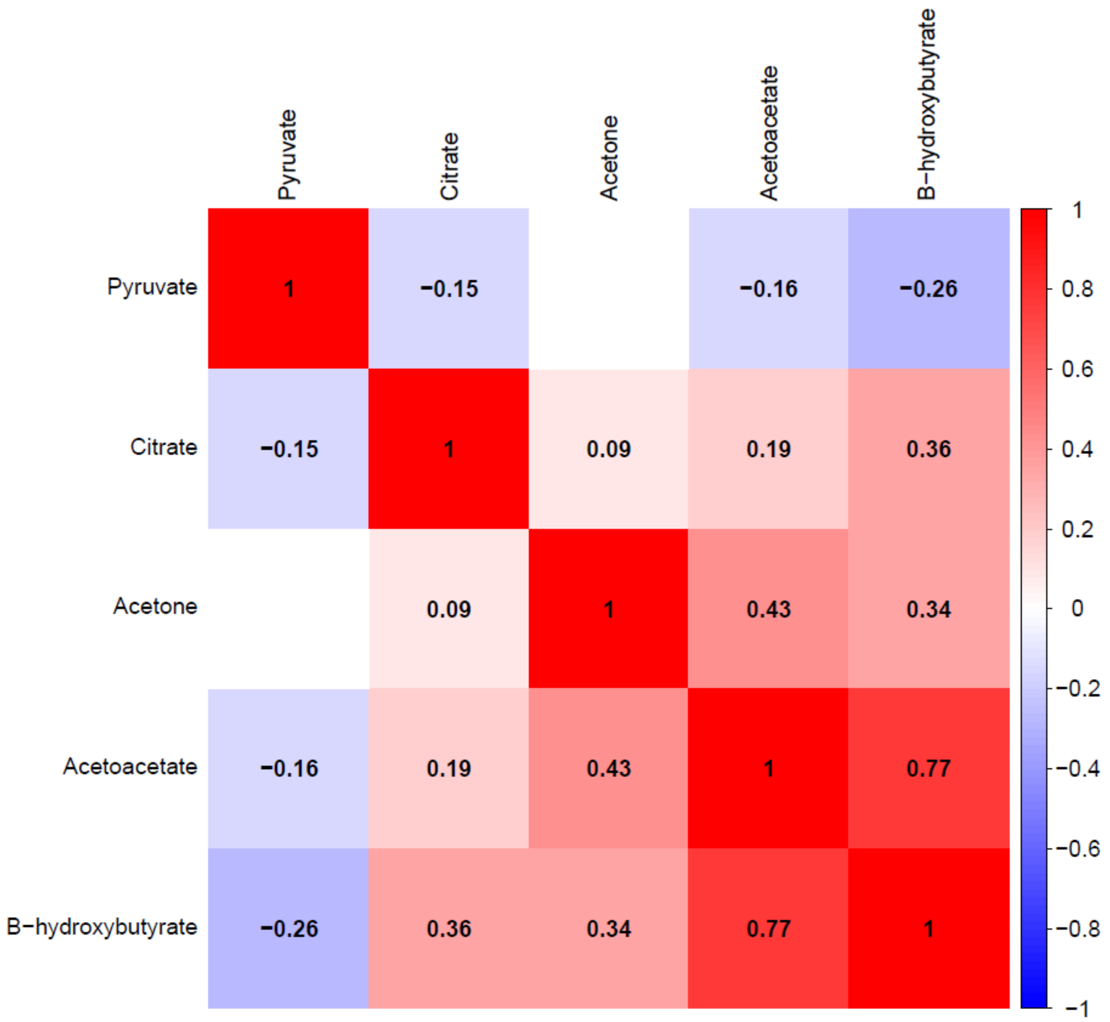

Supplementary Figure 1. Heatmap of correlations among acetone, acetoacetate, β-hydroxybutyrate, pyruvate, and citrate. Color represents the direction of the correlations (red-positive and blue-negative). Only significant correlations are shown.

© 2024. Gutierrez-Tordera L. et al. Published online at http://www.aginganddisease.org/EN/10.14336/AD.2024.0754

SUPPLEMENTARY DATA

Supplementary Table 2. Association of ketone bodies, pyruvate, and citrate, with white matter grade and ventricular grade levels.

Variable	Metabolite	Model 1 β (95% CI)	P value	P_{adj}	Model 2 β (95% CI)	P value	P_{adj}	Model 3 β (95% CI)	P value	P_{adj}
White matter grade	**Ketone bodies score**	0.015 (-0.06, 0.09)	0.687	0.817	0.015 (-0.063, 0.093)	0.708	0.817	NA	NA	NA
	**Acetone**	-0.021 (-0.087, 0.045)	0.527	0.817	-0.036 (-0.103, 0.031)	0.282	0.705	-0.038 (-0.105, 0.029)	0.262	0.705
	**AcAc**	-0.02 (-0.095, 0.055)	0.603	0.817	-0.018 (-0.093, 0.057)	0.643	0.817	-0.032 (-0.112, 0.048)	0.436	0.817
	**BHB**	0.004 (-0.072, 0.08)	0.908	0.908	0.005 (-0.073, 0.083)	0.892	0.908	0.035 (-0.048, 0.118)	0.408	0.817
	**Pyruvate**	-0.079 (-0.168, 0.01)	0.084	0.645	-0.078 (-0.167, 0.011)	0.086	0.645	NA	NA	NA
	**Citrate**	-0.062 (-0.149, 0.025)	0.165	0.705	-0.054 (-0.141, 0.033)	0.225	0.705	NA	NA	NA
Ventricular grade	**Ketone bodies score**	0.026 (-0.041, 0.093)	0.444	0.636	0.024 (-0.045, 0.093)	0.491	0.636	NA	NA	NA
	**Acetone**	-0.05 (-0.108, 0.008)	0.090	0.309	-0.049 (-0.108, 0.01)	0.103	0.309	-0.054 (-0.114, 0.006)	0.077	0.309
	**AcAc**	0.021 (-0.045, 0.087)	0.539	0.636	0.018 (-0.049, 0.085)	0.594	0.636	0.011 (-0.06, 0.082)	0.759	0.759
	**BHB**	0.022 (-0.045, 0.089)	0.513	0.636	0.02 (-0.049, 0.089)	0.565	0.636	0.039 (-0.035, 0.113)	0.309	0.579
	**Pyruvate**	-0.121 (-0.2, -0.042)	**0.003****	**0.023***	-0.124 (-0.203, -0.045)	**0.002****	**0.023***	NA	NA	NA
	**Citrate**	-0.047 (-0.124, 0.03)	0.232	0.579	-0.042 (-0.119, 0.035)	0.289	0.579	NA	NA	NA

NOTE. β represents the score change per standard deviation log metabolite. Model 1 was adjusted for age, sex, race, recruiting center, education level, combined family income, and APOE ε4; Model 2 further adjusted for physical activity, BMI, BMI^2, alcohol intake, alcohol $intake^2$, fasting time, C-reactive protein, albumin, hypertension and diabetes diagnose; Model 3 additionally included mutual ketone body adjustment. FDR was applied to correct for multiple comparisons (P_{adj}). *P value <.05, **P value <.01. ABBREVIATIONS. AcAc, acetoacetate, BHB, B-hydroxybutyrate; CI, confidence interval; BMI, body mass index; FDR, false discovery rate.

© 2024. Gutierrez-Tordera L. et al. Published online at http://www.aginganddisease.org/EN/10.14336/AD.2024.0754

SUPPLEMENTARY DATA

Supplementary Table 3. Association of ketone bodies, pyruvate, and citrate levels with incident dementia.

Metabolite	Model 1 HR (95% CI)	P value	P_{adj}	Model 2 HR (95% CI)	P value	P_{adj}	Model 3 HR (95% CI)	P value	P_{adj}	Model 4 HR (95% CI)	P value	P_{adj}
**Ketone bodies score**	0.955 (0.777, 1.174)	0.662	0.812	0.882 (0.695, 1.118)	0.299	0.779	0.863 (0.676, 1.103)	0.240	0.721	NA	NA	NA
**Acetone**	1.046 (0.919, 1.192)	0.496	0.812	1.026 (0.9, 1.17)	0.696	0.812	1.022 (0.889, 1.176)	0.760	0.833	1.037 (0.908, 1.184)	0.590	0.812
**AcAc**	0.978 (0.796, 1.202)	0.833	0.833	0.954 (0.792, 1.148)	0.615	0.812	0.932 (0.775, 1.12)	0.453	0.812	0.955 (0.773, 1.18)	0.670	0.812
**BHB**	0.975 (0.802, 1.187)	0.803	0.833	0.919 (0.74, 1.142)	0.447	0.812	0.894 (0.712, 1.122)	0.334	0.779	0.93 (0.746, 1.16)	0.522	0.812
**Pyruvate**	0.876 (0.785, 0.978)	**0.018***	0.077	0.847 (0.759, 0.945)	**0.003****	0.060	0.867 (0.774, 0.971)	**0.013***	0.073	NA	NA	NA
**Citrate**	0.942 (0.893, 0.995)	**0.031***	0.110	0.932 (0.882, 0.986)	**0.014***	0.073	0.931 (0.88, 0.985)	**0.013***	0.073	NA	NA	NA

NOTE. Hazard ratios represent risk per standard deviation log (mmol/L) metabolite. Model 1 was adjusted for age, sex, race, recruiting center, education level, combined family income, and APOE ε4; Model 2 further adjusted for physical activity, BMI, BMI^2, alcohol intake, alcohol $intake^2$, fasting time, C-reactive protein, albumin, physical activity, hypertension and diabetes diagnosis; Model 3 included the same confounders as Model 2 and further adjusted for suggested MCI condition; Model 4 additionally included mutual ketone body adjustment. FDR was applied to correct for multiple comparisons (P_{adj}). *P value <.05, **P value <.01. ABBREVIATIONS. AcAc, acetoacetate; BHB, B-hydroxybutyrate; HR, hazard ratio; CI, confidence interval; BMI, body mass index; FDR, false discovery rate.

Supplementary Table 4. Sensitivity analysis of the association between ketone bodies, pyruvate, and citrate, and cognitive function score trajectories through year 11.

Test	Metabolite	Model 1 β (95% CI)	P value	P_{adj}	Model 2 β (95% CI)	P value	P_{adj}	Model 3 β (95% CI)	P value	P_{FDR}
**3MSE**	**Ketone bodies score**	-0.085 (-0.139, -0.03)	**0.003****	**0.011***	-0.079 (-0.133, -0.024)	**0.005****	**0.015***	NA	NA	NA
	**Acetone**	0.029 (-0.025, 0.082)	0.294	0.339	0.021 (-0.033, 0.075)	0.454	0.458	0.02 (-0.034, 0.075)	0.458	0.458
	**AcAc**	-0.057 (-0.113, -0.001)	**0.045***	0.080	-0.054 (-0.109, 0.002)	0.060	0.090	-0.086 (-0.15, -0.023)	**0.008****	**0.020***
	**BHB**	-0.099 (-0.153, -0.044)	**<0.001*****	**<0.001*****	-0.095 (-0.149, -0.04)	**0.001****	**0.005****	-0.096 (-0.152, -0.041)	**0.001****	**0.005****
	**Pyruvate**	0.038 (-0.013, 0.089)	0.145	0.181	0.043 (-0.008, 0.094)	0.102	0.139	NA	NA	NA
	**Citrate**	0.039 (0.003, 0.075)	**0.032***	0.069	0.036 (0, 0.072)	**0.048***	0.080	NA	NA	NA
**DSST**	**Ketone bodies score**	0.005 (-0.038, 0.048)	0.833	0.975	0.006 (-0.037, 0.049)	0.800	0.975	NA	NA	NA
	**Acetone**	0.018 (-0.023, 0.058)	0.396	0.605	0.018 (-0.023, 0.059)	0.388	0.605	0.018 (-0.024, 0.059)	0.403	0.605
	**AcAc**	0.029 (-0.017, 0.074)	0.216	0.605	0.029 (-0.016, 0.075)	0.207	0.605	0.046 (-0.004, 0.096)	0.070	0.350
	**BHB**	-0.002 (-0.045, 0.041)	0.930	0.975	-0.002 (-0.045, 0.042)	0.945	0.975	0.001 (-0.043, 0.044)	0.975	0.975
	**Pyruvate**	0.073 (0.03, 0.115)	**0.001****	**0.008****	0.076 (0.034, 0.119)	**<0.001*****	**<0.001*****	NA	NA	NA
	**Citrate**	0.018 (-0.013, 0.049)	0.262	0.605	0.017 (-0.014, 0.048)	0.285	0.605	NA	NA	NA

NOTE. β represents the metabolite:time interaction estimate (score change per year per standard deviation log metabolite). Model 1 was adjusted for age, sex, race, recruiting center, education level, combined familiy income, and APOE ε4; Model 2 further adjusted for physical activity, BMI, BMI^2, alcohol intake, alcohol $intake^2$, C-reactive protein, albumin, hypertension, and diabetes diagnose; Model 3 additionally included mutual ketone body adjustment. FDR was applied to correct for multiple comparisons (P_{adj}). *P value <.05, **P value <.01, ***P value <.001. ABBREVIATIONS. 3MSE, modified mini-mental state examination; DSST, digital symbol substitution test; AcAc, acetoacetate, BHB, B-hydroxybutyrate; CI, confidence interval; BMI, body mass index; FDR, false discovery rate.

© 2024. Gutierrez-Tordera L. et al. Published online at http://www.aginganddisease.org/EN/10.14336/AD.2024.0754

Supplementary Table 5. Sensitivity analysis of the association of ketone bodies, pyruvate, and citrate, with white matter grade and ventricular grade levels.

Variable	Metabolite	Model 1 β (95% CI)	P value	P_{adj}	Model 2 β (95% CI)	P value	P_{adj}	Model 3 β (95% CI)	P value	P_{adj}
White matter grade	Ketone bodies score	0.025 (-0.05, 0.1)	0.519	0.831	0.033 (-0.045, 0.111)	0.412	0.831	NA	NA	NA
	Acetone	-0.022 (-0.086, 0.042)	0.501	0.831	-0.034 (-0.099, 0.031)	0.304	0.831	-0.038 (-0.104, 0.028)	0.258	0.831
	AcAc	-0.018 (-0.091, 0.055)	0.635	0.831	-0.012 (-0.086, 0.062)	0.746	0.831	-0.031 (-0.11, 0.048)	0.441	0.831
	BHB	0.014 (-0.062, 0.09)	0.722	0.831	0.022 (-0.057, 0.101)	0.578	0.831	0.053 (-0.031, 0.137)	0.216	0.831
	Pyruvate	-0.002 (-0.098, 0.094)	0.968	0.968	-0.014 (-0.11, 0.082)	0.776	0.831	NA	NA	NA
	Citrate	-0.064 (-0.15, 0.022)	0.145	0.831	-0.052 (-0.138, 0.034)	0.238	0.831	NA	NA	NA
Ventricular grade	Ketone bodies score	0.02 (-0.048, 0.088)	0.570	0.676	0.022 (-0.049, 0.093)	0.537	0.676	NA	NA	NA
	Acetone	-0.048 (-0.106, 0.01)	0.107	0.399	-0.045 (-0.104, 0.014)	0.133	0.399	-0.05 (-0.109, 0.009)	0.102	0.399
	AcAc	0.018 (-0.048, 0.084)	0.598	0.676	0.018 (-0.048, 0.084)	0.593	0.676	0.011 (-0.06, 0.082)	0.758	0.758
	BHB	0.017 (-0.052, 0.086)	0.631	0.676	0.02 (-0.051, 0.091)	0.588	0.676	0.038 (-0.038, 0.114)	0.326	0.611
	Pyruvate	-0.09 (-0.175, -0.005)	**0.038***	0.315	-0.089 (-0.175, -0.003)	**0.042***	0.315	NA	NA	NA
	Citrate	-0.055 (-0.132, 0.022)	0.161	0.403	-0.049 (-0.127, 0.029)	0.220	0.471	NA	NA	NA

NOTE. β represents the score change per standard deviation log metabolite. Model 1 was adjusted for age, sex, race, recruiting center, education level, combined family income, and APOE ε4; Model 2 further adjusted for physical activity, BMI, BMI^2, alcohol intake, alcohol $intake^2$, C-reactive protein, albumin, hypertension and diabetes diagnose; Model 3 additionally included mutual ketone body adjustment. FDR was applied to correct for multiple comparisons (P_{adj}). *P value <.05, **P value <.01. ABBREVIATIONS. AcAc, acetoacetate, BHB, B-hydroxybutyrate; CI, confidence interval; BMI, body mass index; FDR, false discovery rate.

Supplementary Table 6. Sensitivity analysis of the association of ketone bodies, pyruvate, and citrate levels with incident dementia.

Metabolite	Model 1 HR (95% CI)	P value	P_{adj}	Model 2 HR (95% CI)	P value	P_{adj}	Model 3 HR (95% CI)	P value	P_{adj}	Model 4 HR (95% CI)	P value	P_{adj}
Ketone bodies score	0.971 (0.786, 1.199)	0.782	0.864	0.907 (0.716, 1.148)	0.416	0.777	0.884 (0.691, 1.132)	0.329	0.777	NA	NA	NA
Acetone	1.059 (0.923, 1.216)	0.413	0.777	1.042 (0.907, 1.196)	0.561	0.777	1.036 (0.895, 1.199)	0.635	0.777	1.05 (0.912, 1.185)	0.498	0.777
AcAc	0.983 (0.796, 1.213)	0.873	0.917	0.959 (0.793, 1.16)	0.666	0.777	0.936 (0.775, 1.13)	0.489	0.777	0.952 (0.764, 1.187)	0.663	0.777
BHB	0.992 (0.811, 1.213)	0.937	0.937	0.946 (0.762, 1.174)	0.615	0.777	0.918 (0.73, 1.153)	0.461	0.777	0.952 (0.764, 1.187)	0.663	0.777
Pyruvate	0.894 (0.792, 1.009)	0.070	0.426	0.865 (0.767, 0.975)	**0.017***	0.367	0.891 (0.788, 1.008)	0.066	0.426	NA	NA	NA
Citrate	0.947 (0.884, 1.015)	0.122	0.426	0.941 (0.873, 1.014)	0.112	0.426	0.94 (0.871, 1.014)	0.112	0.426	NA	NA	NA

NOTE. Hazard ratios represent risk per standard deviation log (mmol/L) metabolite. Model 1 was adjusted for age, sex, race, recruiting center, education level, combined family income, and APOE ε4; Model 2 further adjusted for physical activity, BMI, BMI^2, alcohol intake, alcohol $intake^2$, C-reactive protein, albumin, physical activity, hypertension and diabetes diagnosis; Model 3 included the same confounders as Model 2 and further adjusted for suggested MCI condition; Model 4 additionally included mutual ketone body adjustment. FDR was applied to correct for multiple comparisons (P_{adj}). *P value <.05, **P value <.01. ABBREVIATIONS. AcAc, acetoacetate; BHB, B-hydroxybutyrate; HR, hazard ratio; CI, confidence interval; BMI, body mass index; FDR,

© 2024. Gutierrez-Tordera L. et al. Published online at http://www.aginganddisease.org/EN/10.14336/AD.2024.0754

false discovery rate.

Supplementary Table 7. Sensitivity analysis of the association of ketone bodies, pyruvate, and citrate levels with dementia-related mortality.

Metabolite	Model 1 HR (95% CI)	P value	P_{adj}	Model 2 HR (95% CI)	P value	P_{adj}	Model 3 HR (95% CI)	P value	P_{adj}
**Ketone bodies score**	1.363 (1.154, 1.609)	**<0.001 *** **	**0.002** **	1.282 (1.072, 1.534)	**0.007** **	**0.025***	NA	NA	NA
**Acetone**	1.048 (0.881, 1.247)	0.595	0.638	1.04 (0.875, 1.236)	0.655	0.655	1.053 (0.911, 1.217)	0.488	0.563
**AcAc**	1.199 (0.925, 1.555)	0.170	0.313	1.151 (0.955, 1.387)	0.140	0.300	1.29 (0.911, 1.217)	0.488	0.563
**BHB**	1.378 (1.159, 1.637)	**<0.001 *** **	**0.002** **	1.303 (1.084, 1.565)	**0.005** **	**0.024***	1.29 (1.063, 1.566)	**0.010***	**0.030***
**Pyruvate**	0.929 (0.831, 1.039)	0.199	0.313	0.883 (0.773, 1.009)	0.068	0.170	NA	NA	NA
**Citrate**	1.252 (0.733, 2.136)	0.411	0.560	1.383 (0.834, 2.293)	0.209	0.313	NA	NA	NA

NOTE. Hazard ratios represent risk per standard deviation log (mmol/L) metabolite. Model 1 was adjusted for age, sex, race, recruiting center, education level, combined family income, and APOE ε4; Model 2 further adjusted for physical activity, BMI, BMI^2, alcohol intake, alcohol $intake^2$, C-reactive protein, albumin, physical activity, hypertension and diabetes diagnosis; Model 3 additionally included mutual ketone body adjustment. FDR was applied to correct for multiple comparisons (P_{adj}). *P value <.05. ABBREVIATIONS. AcAc, acetoacetate; BHB, B-hydroxybutyrate; HR, hazard ratio; CI, confidence interval; BMI, body mass index; FDR, false discovery rate.

Supplementary Table 8. Associations of ketone bodies, pyruvate, and citrate with cognitive function score trajectories (3MSE) through year 11 in participants aged 71 or younger and in older participants.

Age	Metabolite	Model 1 β (95% CI)	P value	P_{adj}	Model 2 β (95% CI)	P value	P_{adj}	Model 3 β (95% CI)	P value	P_{adj}
≤71	Ketone bodies score	-0.041 (-0.099, 0.016)	0.155	0.332	-0.024 (-0.081, 0.033)	0.413	0.688	NA	NA	NA
	Acetone	0.166 (0.106, 0.226)	**<0.001*** **	**<0.001*** **	0.176 (0.115, 0.236)	**<0.001*** **	**<0.001 *** **	0.175 (0.115, 0.236)	**<0.001*** **	**<0.001*** **
	AcAc	-0.007 (-0.08, 0.067)	0.862	0.951	0.005 (-0.068, 0.078)	0.888	0.951	0.001 (-0.075, 0.076)	0.985	0.985
	BHB	-0.061 (-0.117, -0.005)	**0.033***	0.124	-0.046 (-0.101, 0.01)	0.107	0.283	-0.034 (-0.09, 0.022)	0.239	0.448
	Pyruvate	0.016 (-0.04, 0.073)	0.575	0.784	0.021 (-0.035, 0.077)	0.459	0.689	NA	NA	NA
	Citrate	0.138 (-0.033, 0.31)	0.113	0.283	0.031 (-0.14, 0.202)	0.723	0.904	NA	NA	NA
>71	Ketone bodies score	-0.061 (-0.157, 0.035)	0.211	0.227	-0.075 (-0.171, 0.02)	0.122	0.180	NA	NA	NA
	Acetone	-0.123 (-0.211, -0.035)	**0.006** **	**0.020***	-0.142 (-0.23, -0.053)	**0.002** **	**0.010***	-0.142 (-0.231, -0.054)	**0.002** **	**0.010***
	AcAc	-0.113 (-0.195, -0.03)	**0.007** **	**0.020***	-0.111 (-0.193, -0.03)	**0.008** **	**0.020***	-0.158 (-0.257, -0.059)	**0.002** **	**0.010***
	BHB	-0.057 (-0.154, 0.04)	0.248	0.248	-0.075 (-0.171, 0.022)	0.130	0.180	-0.095 (-0.193, 0.004)	0.059	0.126
	Pyruvate	0.052 (-0.029, 0.132)	0.212	0.227	0.061 (-0.02, 0.141)	0.140	0.180	NA	NA	NA

© 2024. Gutierrez-Tordera L. et al. Published online at http://www.aginganddisease.org/EN/10.14336/AD.2024.0754

	Citrate	0.032 (-0.011, 0.075)	0.144	0.180	0.033 (-0.01, 0.076)	0.127	0.180	NA	NA	NA

NOTE. β represents the metabolite:time interaction estimate (score change per year per standard deviation log metabolite). Model 1 was adjusted for age, sex, race, recruiting center, educational level, combined family income, and APOE ε4; Model 2 further adjusted for physical activity, BMI, BMI^2, alcohol intake, alcohol $intake^2$, fasting time, C-reactive protein, albumin, hypertension and diabetes diagnosis; Model 3 additionally included mutual ketone body adjustment. FDR was applied to correct for multiple comparisons (P_{adj}). *P value <.05, **P value <.01, ***P value <.001.

© 2024. Gutierrez-Tordera L. et al. Published online at http://www.aginganddisease.org/EN/10.14336/AD.2024.0754
